# Myelination in Multiple Sclerosis Lesions Is Associated with Regulation of Bone Morphogenetic Protein 4 and Its Antagonist Noggin

**DOI:** 10.3390/ijms20010154

**Published:** 2019-01-03

**Authors:** Kim Harnisch, Sarah Teuber-Hanselmann, Nicole Macha, Fabian Mairinger, Lena Fritsche, Daniel Soub, Edgar Meinl, Andreas Junker

**Affiliations:** 1 Institute of Neuropathology, University Hospital Essen, D-45147 Essen, Germany; Kim.Harnisch@uni-wh.de (K.H.); sarah.teuber@uk-essen.de (S.T.-H.); Nicole.Macha@uk-essen.de (N.M.); lena.fritsche1@gmail.com (L.F.); daniel.soub@mail.de (D.S.); 2 Institute of Pathology, University Hospital Essen, D-45147 Essen, Germany; Fabian.Mairinger@uk-essen.de; 3 Institute of Clinical Neuroimmunology, University Hospital and Biomedical Center, Ludwig-Maximilians-Universität München, D-82152 Martinsried, Germany; Edgar.Meinl@med.uni-muenchen.de

**Keywords:** multiple sclerosis, remyelination, remyelination block, bone morphogenetic protein 4, BMP4, Noggin

## Abstract

Remyelination is a central aspect of new multiple sclerosis (MS) therapies, in which one aims to alleviate disease symptoms by improving axonal protection. However, a central problem is mediators expressed in MS lesions that prevent effective remyelination. Bone morphogenetic protein4 (BMP4) inhibits the development of mature oligodendrocytes in cell culture and also blocks the expression of myelin proteins. Additionally, numerous studies have shown that Noggin (SYM1)—among other physiological antagonists of BMP4—plays a prominent role in myelin formation in the developing but also the adult central nervous system. Nonetheless, neither BMP4 nor Noggin have been systematically studied in human MS lesions. In this study, we demonstrated by transcript analysis and immunohistochemistry that BMP4 is expressed by astrocytes and microglia/macrophages in association with inflammatory infiltrates in MS lesions, and that astrocytes also express BMP4 in chronic inactive lesions that failed to remyelinate. Furthermore, the demonstration of an increased expression of Noggin in so-called shadow plaques (i.e., remyelinated lesions with thinner myelin sheaths) in comparison to chronically inactive demyelinated lesions implies that antagonizing BMP4 is associated with successful remyelination in MS plaques in humans. However, although BMP4 is strongly overexpressed in inflammatory lesion areas, its levels are also elevated in remyelinated lesion areas, which raises the possibility that BMP4 signaling itself may be required for remyelination. Therefore, remyelination might be influenced by a small number of key factors. Manipulating these molecules, i.e., BMP4 and Noggin, could be a promising therapeutic approach for effective remyelination.

## 1. Introduction

A major problem of multiple sclerosis (MS) is the inadequate remyelination of its lesions [1]. If this occurs at all, it is mainly in the early stages of the disease and particularly in the peripheral areas of the lesions [2]. Remyelination has axon-protective effects [3]. In addition to restoring nerve conductivity, it shields the axon from the deleterious effects of the surrounding environment, and metabolic interactions with oligodendrocytes are ensured [4]. In contrast, areas of demyelination have an increased number of damaged axons [5]. Axonal degeneration progresses over time and manifests itself in the increasing disability of patients [6]. These observations allow for one to draw the conclusion that the axonal degeneration, which accumulates during the course of multiple sclerosis, can be attributed at least partly to defective remyelination. 

Demyelination of chronic lesions is a major feature in the chronic stage of the disease, regardless of whether they are cortical or white matter lesions [7,8]. 

One reason for defective remyelination is the disturbed differentiation of the oligodendroglial precursor cells towards mature, myelin-forming oligodendrocytes [9,10,11]. Oligodendrocyte precursor cells are present in all activity stages of the lesions, i.e., from inflammatory lesions to chronic lesions with only minimal inflammatory changes [12], but differentiation to mature oligodendrocytes with the formation of myelin sheaths occurs either not at all or only insufficiently. Several factors have been identified that may contribute to the failure of oligodendroglial progenitor cells to fully mature, referred to as a remyelination block. Among these, oligodendrocyte-inhibiting factors of the Notch group, the Wnt-group, or factors such as PSA-NCAM, LINGO-1, or hyaluronic acid with TLR2 receptor have been identified in human MS tissue [13]. Furthermore, the family of bone morphogenetic proteins (BMPs) is able to influence oligodendroglial maturation and myelin protein expression [14].

In a mouse MS model, the experimental autoimmune encephalomyelitis (EAE), in which C57BL6 mice are immunized with MOG_35–55_ peptide, the expression of BMPs, which belong to the TGF-beta-superfamily, has been shown to be increased in inflammatory demyelinating lesions [15]. In particular, bone morphogenetic protein4 (BMP4) was found to be highly upregulated. The findings that BMP4 is upregulated in demyelinating lesions and that it is capable of inhibiting oligodendroglial maturation and myelin protein expression support our hypothesis that BMP4 might be one key factor for remyelination failure. Therefore, antagonizing BMP4 via Noggin might be able to induce effective (re-)myelination, offering a new therapeutic approach for MS patients.

Nonetheless, BMP4 expression has not yet been adequately described in the course of lesion development in human MS lesions. RNA transcripts of BMP4 have only been identified in MS lesions of two MS patients in a single study [16] Against this background, we investigated the distribution pattern of BMP4 expression and the expression of the BMP4 antagonist Noggin in MS lesions of varying inflammatory activity and in remyelinated lesion areas.

## 2. Results

### 2.1. BMP4 Is Upregulated in MS-Lesions

**Figure 1:**
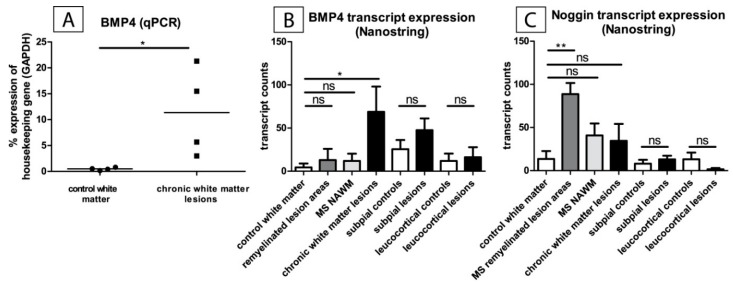
*Bone morphogenetic protein4 (BMP4)* expression and *Noggin* expression in multiple sclerosis lesions. (**A**) *BMP4* is upregulated in chronic white matter lesions. The values were obtained by qPCR from frozen tissue RNA from four different multiple sclerosis (MS) patients and four controls. *Glyceraldehyde 3-phosphate dehydrogenase (GAPDH)* was used as a housekeeping gene. The Mann-Whitney rank sum test was applied for statistical analysis. (* *p* < 0.05). (**B**,**C**) Upregulation of *BMP4* in chronic white matter MS lesions and upregulation of *BMP4* antagonist *Noggin* in remyelinated lesion areas. The results were obtained using nanostring technology from RNA obtained from macrodissected lesion areas of formalin-fixed and paraffin-embedded autopsy (FFPE) tissue. *GAPDH* and *beta-2 microglobulin (B2M)* served as housekeeping genes for comparison. The point of reference for the statistical analysis (Mann-Whitney rank sum test) was always the corresponding control tissue of the white or grey substance (* *p* < 0.05 (**B**, control white matter: *n* = 8, remyelinated lesion areas: *n* = 5, MS NAWM: *n* = 7, chronic white matter lesions: *n* = 9, subpial controls: *n* = 8, subpial lesions: *n* = 11, leukocortical controls: *n* = 8, leukocortical lesions: *n* = 9) and ** *p* < 0.01 (**C**, control white matter: *n* = 8, remyelinated lesion areas: *n* = 5, MS NAWM: *n* = 7, chronic white matter lesions: *n* = 9, subpial controls: *n* = 8, subpial lesions: *n* = 11, leukocortical controls: *n* = 8, leukocortical lesions: *n* = 9), mean + SEM).

In a pilot trial, the upregulation of *BMP4* mRNA levels in chronic inactive MS lesions as compared to control brain tissue was demonstrated in frozen sections of four MS patients and four controls using qPCR (Figure 1A).

In order to evaluate the expression of *BMP4* and the BMP4-antagonizing factor *Noggin (Sym1*) in diverse MS lesions, different areas of grey and white matter lesions, as well as NAWM (normal appearing white matter), NAGM (normal appearing grey matter), and control tissue were macrodissected. The transcript levels of these genes were then determined by nanostring technology (Figure 1B,C). We found an upregulation of *BMP4* in chronic-inactive lesions when compared to controls (Figure 1B). In addition, there was a non-significant tendency to overexpression in subpial lesion areas as compared to control tissue (Figure 1B). In contrast, only low levels of Noggin were observed in all lesion areas and controls. The only exceptions were remyelinated lesion areas i.e., shadow plaques, which showed a significantly increased expression of Noggin (Figure 1C).

### 2.2. BMP4 Is Expressed in Astrocytes and Microglial Cells/Macrophages and Its Upregulation Is Associated with Inflammatory Infiltrates

**Figure 2:**
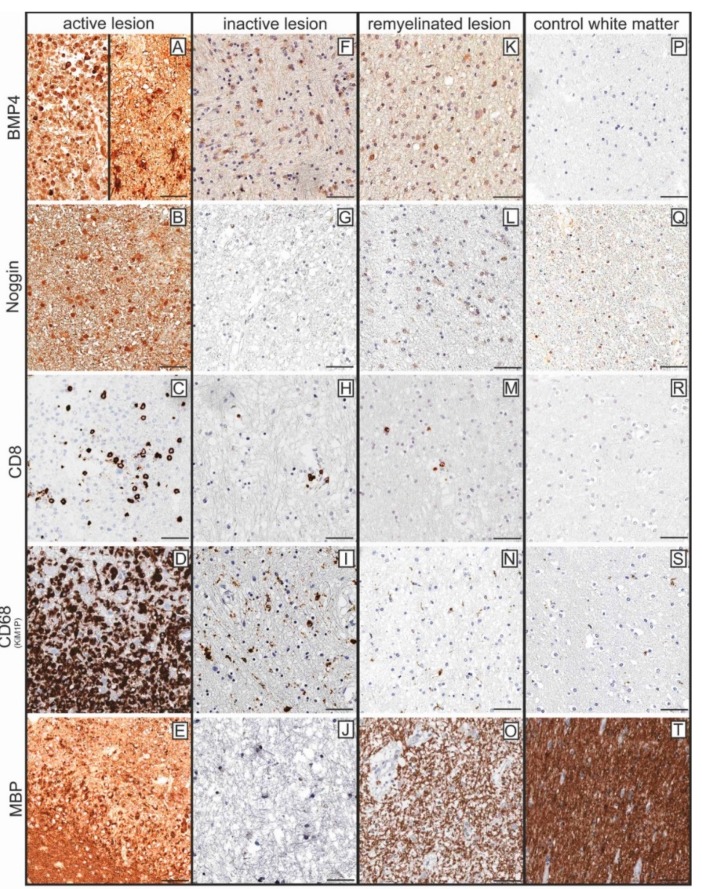
Comprehensive immunohistochemical investigation of the white matter pathology of BMP4 and its antagonist Noggin (representative results). (**A**–**E**) inflammatory (early active*) lesion: BMP4 (**A**) is strongly expressed in foamy macrophages (left) in the lesion. In the lesion rim (right), astrocytic cells with clear overexpression of BMP4 are frequently seen. Increased expression of the BMP4 antagonist Noggin in active lesions (**B**). The inflammatory infiltrate was shown here with the T cell marker CD8 (**C**), which detects more than 50% of the T-cells in the infiltrate (Supplementary Figure S1). The foamy macrophages in the lesion are marked positively in the staining against CD68 (KiM1P) (**D**). In the marginal area of the lesion, the staining against the myelin protein MBP (**E**) shows intact myelin sheaths (lower area). In addition, some macrophages are present in the infiltrate (middle region) in whose cytoplasm myelin degradation products are recognizable (in this case MBP; CNP also contained in macrophages, not shown). (**F**–**J**) chronic inactive lesion: BMP4 (**F**) is expressed in some astrocytes in the lesion center. Noggin is hardly expressed (**G**). In the chronically inactive lesions, only a few T-cells (CD8, **H**) and slightly activated microglia/residual macrophages (CD68 (KiM1P), (**I**) are still present. Myelin sheath are not detectable any more in the lesion centre (MBP, **J**). (**K**–**O**) remyelinated lesion area: BMP4 (**K**), like Noggin (**L**), is expressed in some cell elements in remyelinated lesion areas. Also in these lesion areas there are only few inflammatory infiltrates (CD8, **M**) and only solitary activated microglial cells (CD68 (KiM1P), **N**). In the MBP staining some thin (newly formed) myelin sheaths are stained (**O**). (**P**–**T**) white matter control tissue: No cells are marked positively in staining against BMP4 (**P**). Some cells express Noggin (**Q**). No T-lymphocytes are marked in the parenchyma (**R**). Only solitary microglial cells are detected in staining against CD68 (KiM1P) (**S**). The myelin sheaths are intact (MBP, **T**). (* early active in this context means that, in addition to inflammatory infiltrate in macrophages, small phagocytosed myelin protein fragments (CNP, not shown) could be detected in the lesion [17]); (scale bar = 50 µm).

Immunohistochemistry was performed in order to gain insight into the distribution pattern of BMP4- and Noggin-expressing cells in diverse MS lesions (Figure 2). 

The differences in the expression levels of BMP4 and its antagonist are comparable to those of the transcript level. BMP4 is particularly expressed in foamy macrophages (A, left side) and in reactive astrocytes (A, right side) in the lesion rims of active i.e., inflammatory lesions. In addition, BMP4 expression is elevated in chronically inactive lesion centers (F) and in remyelinated lesion sites (K) as compared to control tissue (P). Noggin is upregulated in active lesions (B) as well, whereas chronically inactive lesion centers show virtually no expression (G). Interestingly, remyelinated lesion sites (L) exhibit a higher expression of Noggin than chronically inactive lesion centers (G). Inflammatory and microglial/macrophagocytic infiltrates as well as the degree of myelination are shown in the corresponding figures (A:C,D,E; F:H,I,J; K:M,N,O; P:R,S,T).

**Figure 3:**
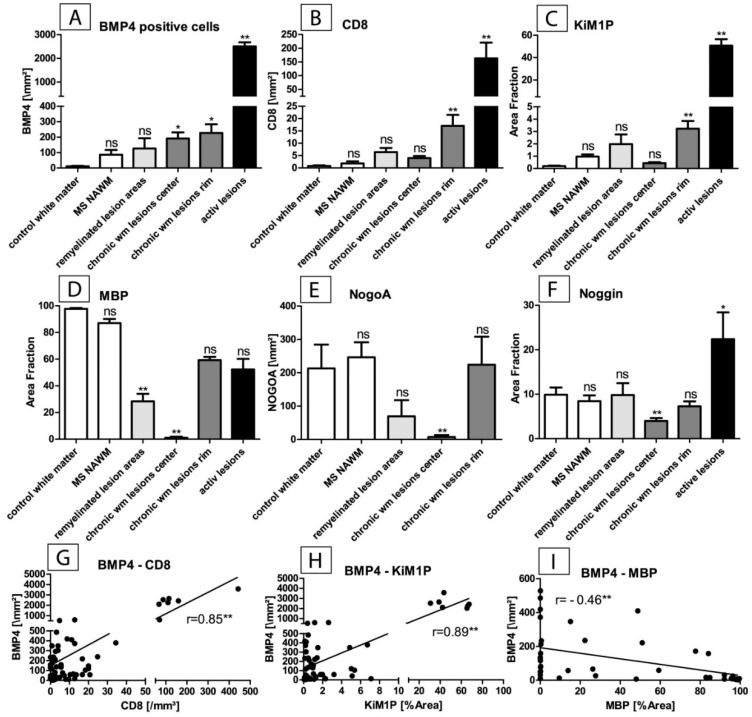
Software-assisted immunohistochemical quantification of BMP4-positive cells associated with the white matter pathology of multiple sclerosis: (**A**) Quantification of immunopositive cells in inflammatory i.e., active lesions, the lesion margin of chronically inactive lesions, the lesion center of chronically inactive lesions, remyelinated lesion sites and normal appearing white matter (NAWM) of MS cases with respect to control tissue (white matter of cases without apparent neurological disease). A significantly increased number of BMP4-positive cells is found in active lesions. A larger number of BMP4-positive cells are also seen in the periphery and center of chronically inactive lesions (** *p* < 0.01, * *p* < 0.05, *n* = 6–18, mean + SEM, ns = not significant). (**B**,**C**) Quantification of CD8-positive T-cells and the microglial/macrophagocytic infiltrate (CD68 (KiM1P) area fraction) in corresponding lesion areas as described under A. Inflammatory T-cellular infiltrate and microglial/macrophagocytic infiltrate are particularly present in active lesions and in the margins of chronically inactive lesions (** *p* < 0.01, *n* = 6–18, mean + SEM, ns = not significant). (**D**,**E**) Quantification of the abundance of myelin sheaths (detected by staining for the myelin protein MBP; MBP area fraction) and of mature oligodendrocytes (NogoA-staining). Compared to control tissue, myelin sheaths (**D**) are reduced particularly in the chronically inactive lesion centers and also in remyelinated lesion sites (** *p* < 0.01, *n* = 6–18, mean + SEM, ns = not significant). The number of mature oligodendrocytes (NogoA-staining, **E**) is significantly reduced only in the lesion centers of chronically inactive lesions. In the remyelinated lesion areas, their number has increased again and there is no significant difference compared to control tissue. (** *p* < 0.01, *n* = 6–18, mean + SEM, ns = not significant). (**F**) Quantification of the BMP4 antagonist Noggin (area fraction). Increased expression is observed in inflammatory lesions. Especially in lesion centers of chronically inactive lesions, one observes a considerably reduced expression of Noggin. There is a marked difference between the lesion centers of chronically inactive lesions and remyelinated lesion sites in which Noggin expression is restored. Both the inflammatory T-cellular infiltrate and the microglial infiltrate correlate with the number of BMP4-positive cells (** *p* < 0.01, *r* = 0.85 (**G**), and *r* = 0.89 (**H**)). A negative correlation between BMP4 expression and myelin sheathing is observed (** *p* < 0.01, *r* = −0.46 (**I**).

We used image processing software (ImageJ) to quantify the number of stained cells (BMP4, T cell subset (CD8), mature oligodendrocytes (NogoA)), and the percentage of stained sectional area (microglia/macrophages (KiM1P staining), myelin protein (MBP staining), and Noggin) (Figure 3).

Quantitative analysis revealed that BMP4 is significantly upregulated, especially in active lesions but also in chronically inactive lesions (3A). Additionally, there was an association with inflammatory (CD8, 3B) and microglia/macrophage infiltrates (KiM1P, 3C), with Pearson’s correlation coefficient values of 0.85 (BMB4-CD8) and 0.89 (BMP4-KiM1P), respectively (3G, H). There was a negative correlation between BMP4 and myelin sheaths (3D), with a Pearson’s correlation coefficient of −0.46 (BMB4-MBP). Interestingly, the immunohistochemical quantification of the BMP4 antagonist Noggin showed a higher protein expression in remyelinated lesion areas as compared to chronically inactive demyelinated lesions (3F).

**Figure 4:**
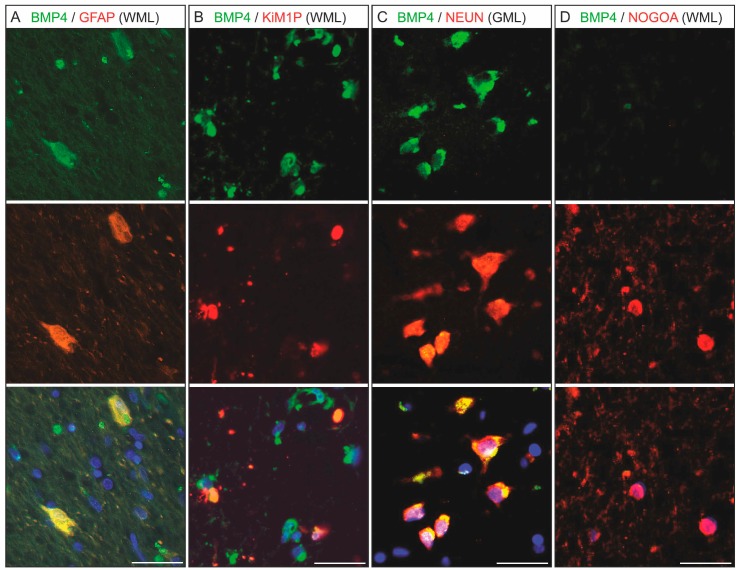
Chronic white matter lesions (WML) or grey matter lesions (GML) with BMP4 expressing cells. The double fluorescence staining of BMP4 (**A**–**D**, green) with GFAP (**A**, red), CD68 (Kim1P) (**B**, red), and NeuN (**C**, red) shows that BMP4 is expressed by astrocytes (**A**), microglia/macrophages (**B**), and neurons (**C**). The NogoA-positive adult oligodendrocytes (**D**, red) do not express BMP4; (scale bar = 25 µm).

We performed immunohistochemical double staining to determine the specific cell types that expressed BMP4 (Figure 4).

We found that BMP4 was expressed in astrocytes (GFAP, A) as well as in microglial cells/macrophages (KiM1P, B) and neurons (NeuN, in grey matter, C). Adult oligodendrocytes did not exhibit any BMP4 expression (NogoA, D). Furthermore, the two astrocytic subtypes described in the literature (A1- and A2-astrocytes) [18] are both capable of expressing BMP4 (Supplementary Figure S2).

## 3. Discussion

Our results show that mRNA and protein levels of the myelin inhibiting factor BMP4 are upregulated in various stages of human MS lesions. Furthermore, we were able to show that BMP4 is expressed in astrocytes and microglial cells/macrophages and that this upregulation is associated with inflammatory infiltrates. The BMP4 antagonist Noggin exhibited a significantly decreased expression in chronically inactive, demyelinated lesion sites as compared to remyelinated lesion sites, and at least on transcript level in control tissue. This indicates that remyelination only takes place in areas in which the effects of BMP4 are at least partially antagonized.

BMP4 belongs to the family of bone morphogenetic proteins (BMPs). BMPs play a role in a variety of biological processes and they are expressed in numerous cell types [19]. For example, BMPs play a role in the regulation of immune processes [20]. BMPs are secretory growth factors that form the largest subgroup of the transforming growth factor beta (TGF-beta) superfamily signal ligands [21]. Binding of secreted BMPs to a hetero-tetrameric receptor complex consisting of one type 1 receptor and one type 2 BMP receptor results in intracellular activation of the SMAD signaling pathway [22]. BMP4, and to a lesser extent BMP6 and BMP7, is upregulated in the EAE mouse model, and it is associated with clinical symptoms and disease progression [15]. It is known that the BMPs 2, 4 and 7 are capable of inducing astroglial differentiation of progenitor cells while simultaneously inhibiting the formation of oligodendrocytes in vitro [23,24] and in vivo [25]. In addition, the overexpression of BMP4 caused inhibition of oligodendrogenesis in Xenopus embryos [26]. Although BMP4 appears to be expressed by a large variety of cells, we were able to show that at least some mature oligodendrocytes do not express BMP4. Furthermore, not only the maturation of oligodendrocytes but also the formation of myelin proteins (PLP, MBP) in maturing oligodendrocytes was inhibited [27]. Our data support this observation, as we were able to show a negative correlation between BMP4 and myelin protein expression (Figure 3I). The inhibition of oligodendroglial progenitor cells is associated with the increased maturation of astroglial progenitor cells into adult astrocytes [23,24]. This observation leads to the hypothesis that BMP4 (in combination with other molecules) may be involved in the formation of “astrocytic scars” in MS lesions [19]. 

A recent study has demonstrated that astrocytes of two different phenotypes may be differentially distributed in MS lesions, and that this appears to depend on the stage of the disease or the inflammatory activity [18]. The C3D-expressing, so-called neurotoxic astrocytic phenotype was observed in active lesions with dense macrophagocytic penetration (A1), while S100a10-positive astrocytes of type A2 (protective phenotype) were found in lesions of different inflammatory activity [18]. Our double-staining technique clearly demonstrated that the expression of BMP4 was not restricted to only one of the two astrocytic phenotypes, but that C3D-positive neurotoxic and S100a10-positive protective astrocytes both expressed BMP4. Since our results indicate that BMP4 is overexpressed in every lesion stage when compared to controls, this observation is not surprising, but it confirms the results of Liddelow and colleagues (Supplementary Figure S2).

Increased BMP4 expression in association with an inflammatory infiltrate, particularly in active lesions, on the one hand and overexpression in the astrocytic scar, i.e., the lesion center of chronic inactive lesions, on the other hand, may result in permanent BMP4 signaling during the entire evolution of the lesion. This uninterrupted BMP4 signalling might, in turn, prevent effective remyelination and thus contribute to the progressive axonal degeneration in the course of the disease.

A variety of extracellular molecules, e.g., chordin, follistatin, gremlin, or Noggin (SYM1) antagonize different BMPs with varying affinity [28,29]. Among the physiological antagonists, Noggin is the one most studied in the central nervous system. Noggin is a glycosylated, homodimeric, secretory glycoprotein [30] that binds with high affinity to BMPs, such as BMP2 and BMP4 [31,32], as well as to BMP5 [32] and BMP7 [33]. With regard to demyelination, Noggin administration induces increased oligodendrocyte differentiation in the cuprizone mouse model [34,35]. We demonstrated an increased expression of the BMP4 antagonist Noggin in remyelinated lesion areas when compared to chronically inactive demyelinated lesion centers. This implies that successful remyelination is associated with the antagonization of BMP4, which subsequently results in the maturation, and possibly formation, of oligodendrocytes. However, the fact that BMP4 is also expressed in remyelinated lesion areas may imply that a certain degree of BMP4 signaling is also necessary for efficient remyelination. This hypothesis is supported by similar findings in animal experiments, which showed that myelination and oligodendrocyte maturation required a certain amount of BMP4 signaling. In those experiments, a complete blockade of BMP4 resulted in the blockade of myelination [36].

The functional effect of antagonizing BMP4 on the time course of lesion development with regard to successful remyelination was not the subject of this study and must be investigated separately in animal studies. It is, however, uncertain whether the complex processes that take place in humans during faulty remyelination can be reliably reproduced in animal models of MS, since these do not reproduce in their entirety the pathophysiological processes of inflammation, demyelination, defective remyelination, axonal damage, and glial scarring in humans [37].

In a review, Kotter describes a window of opportunity during which oligodendrocyte precursor cells can successfully differentiate in an inflammatory demyelinating process [11]. This review describes factors that promote or inhibit remyelination. Among those that support the differentiation of oligodendroglial progenitor cells towards mature, myelin-forming oligodendrocytes are components of the extracellular matrix, such as beta1- or beta5-integrin [38,39,40], and vitronectin [41,42], neurotransmitters that act at glutamate receptors and beta-adrenoreceptors [43,44], as well as the chemokine CXCL12. The latter accelerates the differentiation of oligodendrocyte precursor cells via its receptor CXCR7 in serum-containing medium [45]. Inhibiting factors of oligodendrocyte precursor cells are partly expressed, depending on the inflammatory activity of the lesions. Jagged 1 was detected in astrocytes [46] in the margins of active and chronically active lesions. In addition, Wnt signalling molecules have been identified in chronically active lesions [47], and PSA-NCAM [48] and hyaluronic acid [49] have been described in chronic inactive lesions. These studies demonstrate that there is a complex interaction between factors inhibiting and promoting remyelination before successful remyelination.

The association between BMP4 and inflammatory/macrophagocytic infiltrates suggests that the modulation of the inflammatory activity of lesions with a subsequently altered BMP4 expression has an indirect, positive influence on remyelination. On the other hand, studies indicate that the acute inflammatory response creates an environment that has a positive effect on remyelination. Depletion of macrophages [50] or T-cells in an animal model [51] inhibits efficient remyelination in the CNS, while the induction of acute inflammation promotes remyelination [52]. Also, in our study, the BMP4 antagonist Noggin was highly expressed in active lesions, which may positively influence early remyelination. Systemic anti-inflammatory therapy may thus prevent the formation of the milieu required for remyelination. This demonstrates the therapeutical dilemma of either modulating inflammation or stimulating remyelination. 

Overexpression of BMP4 in MS lesions with various inflammatory activities, coupled with reconstituted Noggin expression in remyelinated lesion areas, shows that remyelination may be influenced by a small number of key factors. Manipulating these molecules, i.e., BMP4 and Noggin, could be promising as a therapeutic approach to remyelination.

## 4. Material and Methods

### 4.1. Human Brain Tissue

The investigations were performed on formalin-fixed and paraffin-embedded autopsy tissue (FFPE) from 16 autopsied MS patients, as well as biopsy material from eight MS patients and from eight age-matched autopsy controls (Table 1). All of the autopsied patients had suffered from long-term chronic multiple sclerosis. The MS biopsies were taken from patients with a suspected brain tumor for diagnostic purposes. Neuropathological examination revealed these lesions to be inflammatory and demyelinating.

The material from autopsied MS patients was obtained from the Netherlands Brain Bank (NBB), where it was evaluated and the diagnosis was confirmed by Andreas Junker. The MS biopsy material, as well as the control tissues, were obtained from the archive of the Institute of Neuropathology of the University Hospital Essen where they had been diagnosed. The clinical history of the patients was evaluated for the study. The study was approved by the ethics committee of the Ethics Commission of the University of Duisburg-Essen (reference: 16-6933-BO). All of the investigations were performed in compliance with relevant laws and institutional guidelines, and they were carried out following the rules of the Declaration of Helsinki of 1964, revised in 2013. 

### 4.2. Histology and Immunohistochemistry

All of the investigations were performed on 1 μm sections. In addition to standard staining with hematoxylin-eosin (HE) (not shown), immunohistochemical staining was performed with antibodies against BMP4, Noggin, CD8, CD3, MBP, NogoA, KiM1P (CD68), and NeuN, according to standard procedures. Pretreatments and antibody dilutions were carried out as described in Table 2. In brief, the endogenous peroxidase activity was first blocked by incubation of the sections in 3% H_2_O_2_ in PBS. This was followed by a blocking step with 10% fetal calf serum in PBS for ten minutes at room temperature, followed by incubation with the primary antibody for one hour at room temperature. They were then incubated with the secondary antibody (fluorescence or biotin labelled antibody). Finally, the conventional immunohistochemical staining was developed with 3,3′-diaminobenzidine (DAB). Cell nucleus counterstaining was performed with hematoxylin (for conventional staining) or with 4′,6-diamidino-2-phenylindole (DAPI) for fluorescence staining. Some sections were stained using the DAKO Autostainer Plus. In these cases, the ZytoChemPlus HRP Polymer System (Mouse/Rabbit) (REF:POLHRP-100) was used for detection. In addition, immunofluorescence double staining was performed with BMP4 + C3D, BMP4 + S100A10, BMP4 + Nogo A, BMP4 + NeuN, and BMP4 + KiM1P (CD68) (Table 2). The anti-CD3 staining gave no evaluable results with the autopsy tissue. Both CD3-positive T-cells and CD8-positive T-cells were detected and evaluated in the biopsy tissue. A comparison of CD3 and CD8 cell counts showed more than 50% of the T-cells were usually positive for CD8 (Supplementary Figure S1). Therefore, in this study, the inflammatory infiltrates were usually quantified using the marker for CD8-T-cells, which also worked well with autopsy tissue).

The stained sections were first digitized using a Leica slide scanner. From the scanned files, five areas with an edge length of 500 µm were extracted from all regions of interest and analyzed using Image J [53]. After adjusting hue, saturation, and brightness, the “color threshold” was adjusted so that colored particles or colored areas could be determined using the “Analyze Particles” function [54]. In white matter, centers of chronically inactive demyelinated lesions, their margins, normal appearing areas, remyelinated lesion sites, early active lesions (with CNP myelin degradation products in macrophages), and corresponding control tissue, if present, were analyzed in each slice. The fact that BMP4 is regularly expressed in neurons (Figure 4) made histomorphological analysis of gray matter lesions impossible in this study.

### 4.3. cDNA Synthesis and Quantitative PCR

The RNA from chronically inactive lesions in frozen material and corresponding frozen controls was gathered in an earlier study [55]. The transcription of RNA into complementary DNA (cDNA) was performed with the high-capacity cDNA reverse transcription kit (Thermo Fischer Scientific, Waltham, MA, USA), according to the manufacturer’s instructions. 200 ng RNA aliquots were used for each reaction (20 µL). The qPCR reaction was performed with the qPCR core kit and uracil-*N*-glycosylase (both from Eurogenetec, Lüttich, Belgium). GAPDH was used as housekeeping gene [54]. Transcripts were analyzed with TaqMan assays (Thermo Fisher Scientific, former Applied Biosystems, Foster City, CA, USA) against BMP4 (Assay number: Hs00370078_m1).

### 4.4. Nanostring

Relevant areas were extracted by macrodissection from 5 µm paraffin sections that had previously been mounted on foil-coated slides (MS patients and controls). Total RNA was obtained using the miRNeasy FFPE kit (Qiagen, Hilden, Germany). From each RNA sample, the relevant transcripts (BMP4, Noggin, GAPDH, B2M) were quantified using nanostring technology [56]. In brief, the total amount of RNA (100 ng) from each sample was hybridized overnight and evaluated using a digital analyzer (NanoString, Seattle, WA, USA). A specially prepared multiplex probe library was used, each with two sequence-specific probes. The capture probe (35–50 bp) was coupled to biotin, and the reporter probe (35–50 bp) to a color code.

### 4.5. Statistical Analysis

GraphPad Prism 5.0 was used for statistical analysis and evaluation. The Mann-Whitney U-Test was used to compare independent groups. The correlation between groups was calculated as Pearson’s *r*. One-way ANOVA for non-parametric data was utilized to compare more than one group with each other. A *p*-value of <0.05 was considered to be statistically significant and <0.01 as highly significant.

## Figures and Tables

**Table 1:** MS autopsy cases and controls.

Case	Age	Sex	Cause of Death	Inflammatory Circumstannces Perimortal
**Multiple Sclerosis—autopsy cases**				
**MS-01**	66	female	cancer metastases in the liver resulting in severe failure of the liver functions	no
**MS-02**	75	female	pneumonia	pneumonia
**MS-03**	68	female	pneumonia	pneumonia
**MS-04**	78	female	stroke	no
**MS-05**	49	male	pneumonia	pneumonia
**MS-06**	55	male	Respiratory insufficiency by pneumonia and urosepsis	pneumonia and urosepsis
**MS-07**	44	male	pneumonia by aspiration	pneumonia
**MS-08**	44	male	multiorgan failure	unkown
**MS-09 + MS-10**	57	female	respiratory insufficiency	(uro)sepsis
**MS-11**	53	male	Euthanasia	unknown
**MS-12**	62	female	cachexia and pulmonary insufficiency	no
**MS-13 + MS-14**	56	female	respiratory insufficiency by Pneumonia	pneumonia
**MS-15**	54	female	heart failure	unknown
**MS-16**	58	male	terminal renal insufficiency	pneumonia
**MS-17**	63	male	pneumonia	pneumonia
**MS-18**	48	female	respiratory failure	Unknown
**Multiple Sclerosis—biopsy cases**				
**MS-19**	48	male		
**MS-20**	46	female		
**MS-21**	57	female		
**MS-22**	36	female		
**MS-23**	47	male		
**MS-24**	43	male		
**MS-25**	61	female		
**MS-26**	46	female		
**Controls—autopsy cases**				
CON-01	46	female	haemorrhagic shock, sepsis	sepsis
CON-02	55	male	aortic dissection	pancreatitis
CON-03	56	female	pulmonary embolism	no
CON-04	56	male	hepatic insufficiency after liver transplantation	no
CON-05	58	female	cardiogenic shock	no
CON-06	63	male	pulmonary insufficiency in pneumonia	pneumonia
CON-07	66	female	multiorgan failure	no
CON-08	68	female	cardiorespiratory insufficiency	no

**Table 2:** Antibodies and staining procedures.

Antigen	Company	Pre-Treatment	Dilution
BMP4, ab39973, rabbit pc	Abcam	citrate	1:60
Nogo A,sc-25660,clone H-300, rabbit pc	SantaCruz Biotechnology	citrate	1:100
CD8, clone C8/144B, mouse mc	DAKO	citrate	1:150
CD3,cloneSP7, mouse mc	DCS	citrate	1:100
C3D, A0063	DAKO	citrate	1:400
S100A10,MA5-15326, clone 4E7E10, mouse mc	ThermoFisher	citrate	1:1000
KiM1P (CD68)	kind gift from Prof. Klapper, Institute for Pathology, Kiel, Germany	none	1:10.000
MBP, REF A0623, rabbit pc	DAKO	none	1:1000
Noggin, ab16054, rabbit pc NeuN, ab104225	Abcam Abcam	Citrate Citrate	1:800 1:1000

## Supplementary Materials

The supplementary materials are available online at http://www.mdpi.com/1422-0067/20/1/154/s1.
