# Supplementary material for: Myelination in Multiple Sclerosis Lesions Is Associated with Regulation of Bone Morphogenetic Protein 4 and Its Antagonist Noggin
Source: Int J Mol Sci. 2019 Jan 3;20(1):154. doi: 10.3390/ijms20010154 (PMC6337410; doi:10.3390/ijms20010154)
Supplement: Supplementary file 1 [file ijms-20-00154-s001.pdf]

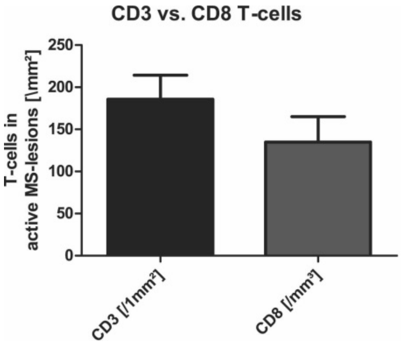

Figure S1. T-cellular infiltration in active MS-lesions. A comparison of the CD3 cell counts with the CD8 cell counts in the active lesions (biopsy MS-tissue) shows that generally more than 50% of the T-cells are positive for CD8. Since no satisfactory staining result could be achieved with the marker for CD3-T-cells in the investigated autopsy tissue (possibly for technical reasons), the well-functioning marker for CD8-T-cells was used for the characterization of the inflammatory infiltrate in this paper.

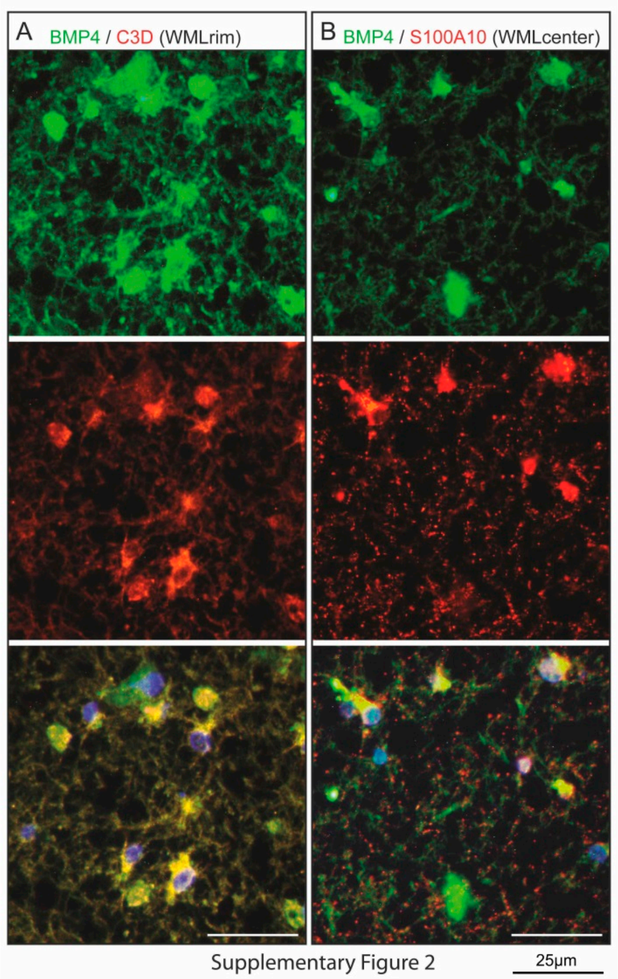

Figure S2. Chronic white matter lesions (WML) with astrocytes expressing BMP4. BMP4 expression in the astrocytic subtypes A1 and A2 described in the literature [17] was examined by immunohistochemical double staining. BMP4 (A, B, green) expression was detected in A1 astrocytes (A, C3D, red) and A2 astrocytes (B, S100A10, red); scale bar = 25µm.
